# The relationship between body temperature, heart rate and respiratory rate in acute patients at admission to a medical care unit

**DOI:** 10.1186/1757-7241-23-S1-A12

**Published:** 2015-07-16

**Authors:** Maria M Jensen, Mikkel Brabrand

**Affiliations:** 1 Emergency Department, Skånes University Hospital, Lund, Sweden; 2 Emergency Department, Sydvestjysk Sygehus, Esbjerg, Denmark

## Background

An increase in body temperature (BT) is followed by an increase in heart rate (HR) and respiratory rate (RR). Only a few studies have explored the magnitude of this increase. These studies all included young healthy study subjects not taking any medicine that influenced the cardiovascular system. We wished to investigate this relationship in a study group more representative of the acute patients we meet in an emergency department.

## Methods

Vital parameters from 4,493 acute patients obtained at admittance to the medical admission unit at Sydvestjysk Sygehus, Esbjerg, were retrospectively extracted from the hospital database. Linear and multiple variable regression analysis was used to calculate the change in HR (ΔHR/°C) and RR (ΔRR/°C) corresponding to variations in BT for the entire study group and after dividing the group in low (<36.4°C), normal (36.4-37.2°C) and high (>37.2°C) BT.

## Results

The study population consisted of 2,219 males and 2,274 females with a mean age of 62.2 ± 19.2 years. The ΔHR/°C and ΔRR/°C for the whole population was 7.2 ± 0.4 beats per minute (bpm) and 1.4 ± 0.1 breaths per minute (brpm). When adjusting for age, oxygen saturation and mean blood pressure, the results were 6.4 ± 0.4 bpm and 1.2 ± 0.1 brpm, respectively. In groups with low, normal and high BT the ΔHR/°C for the three groups were 2.7 ± 1.9, 6.9 ± 1.9 and 7.4 ± 0.9 bpm, respectively. With regard to ΔRR/°C the values were -0.5 ± 0.5, 1.5 ± 0.5 and 2.3 ± 0.3 brpm, respectively.

## Conclusions

The previously most widely cited study on the association between BT and HR was performed in 1951 and reported a ΔHR/°C of 14.7 bpm. Later studies have shown a mean ΔHR/°C of 9.7 bpm. We have been unable to locate any references for the association between BT and RR. However, a ΔRR/°C of 2.0-4.0 brpm seems to be mostly agreed upon in the literature. We found a somewhat lower ΔHR/°C and ΔRR/°C than previously reported, in our population of subjects, adjusting for age, oxygen saturation and mean blood pressure. The highest ΔHR/°C and ΔRR/°C were seen in the groups with the highest BT. We found no significant trends in the groups with low BT.

